# Characteristics associated with subjective and objective measures of treatment success in women undergoing percutaneous tibial nerve stimulation vs sham for accidental bowel leakage

**DOI:** 10.1007/s00192-022-05431-y

**Published:** 2023-01-27

**Authors:** Douglas Luchristt, Benjamin Carper, Sunil Balgobin, Isuzu Meyer, Deborah Myers, Donna Mazloomdoost, Marie Gantz, Uduak Andy, Halina M. Zyczynski, Emily S. Lukacz, On behalf of the Pelvic Floor Disorders Network, Halina M. Zyczynski, Holly E. Richter, Vivian W. Sung, Emily S. Lukacz, Lily A. Arya, David D. Rahn, Anthony G. Visco, Donna Mazloomdoost, Benjamin Carper, Marie G. Gantz

**Affiliations:** 1 Duke University Medical Center, Durham, NC USA; 2 RTI International, Research, Triangle Park, NC USA; 3 University of Texas Southwestern, Dallas, TX USA; 4 University of Alabama at Birmingham, Birmingham, AL USA; 5 Brown University, Women’s & Infants Hospital, Providence, RI USA; 6 Eunice Kennedy Shriver National Institute of Child Health and Human Development, Bethesda, MD USA; 7 University of Pennsylvania, Philadelphia, PA USA; 8 Magee-Womens Research Institute, University of Pittsburgh, Pittsburgh, PA USA; 9 University of California San Diego, La Jolla, CA USA

**Keywords:** Accidental bowel leakage, Fecal incontinence, Percutaneous tibial nerve stimulation, Patient-centered outcomes, St Mark’s score, Treatment success definition

## Abstract

**Introduction and hypothesis:**

In randomized trials both percutaneous tibial nerve stimulation (PTNS) and sham result in clinically significant improvements in accidental bowel leakage (ABL). We aimed to identify subgroups who may preferentially benefit from PTNS in women enrolled in a multicenter randomized trial.

**Methods:**

This planned secondary analysis explored factors associated with success for PTNS vs sham using various definitions: treatment responder using three cutoff points for St. Mark’s score (≥3-, ≥4-, and ≥5-point reduction); Patient Global Impression of Improvement (PGI-I) of ≥ much better; and ≥50% reduction in fecal incontinence episodes (FIEs). Backward logistic regression models were generated using elements with significance of *p*<0.2 for each definition and interaction terms assessed differential effects of PTNS vs sham.

**Results:**

Of 166 women randomized, 160 provided data for at least one success definition. Overall, success rates were 65% (102 out of 158), 57% (90 out of 158), and 46% (73 out of 158) for ≥3-, ≥4-, and ≥5-point St Mark’s reduction respectively; 43% (68 out of 157) for PGI-I; and 48% (70 out of 145) for ≥50% FIEs. Of those providing data for all definitions of success, 77% (109 out of 142) met one success criterion, 43% (61 out of 142) two, and 29% (41 out of 142) all three success criteria. No reliable or consistent factors were associated with improved outcomes with PTNS over sham regardless of definition.

**Conclusions:**

Despite exploring diverse success outcomes, no subgroups of women with ABL differentially responded to PTNS over sham. Success results varied widely across subjective and objective definitions. Further investigation of ABL treatment success definitions that consistently and accurately capture patient symptom burden and improvement are needed.

**Supplementary information:**

The online version contains supplementary material available at 10.1007/s00192-022-05431-y

## Introduction

Accidental bowel leakage (ABL), also known as fecal incontinence, is defined as the involuntary loss of solid or liquid stool from the rectum [1, 2]. Prevalence estimates range from 7 to 20% among community-dwelling adult women, and rates reach up to 70% in nursing home populations [1–3]. The negative impact on psychosocial functioning and quality of life can be devastating, leading to stigmatization, low self-esteem, social isolation, and psychiatric disorders [4, 5]. When conservative options such as lifestyle modifications, pharmacological therapy, and pelvic floor exercises with or without biofeedback are insufficient to control symptoms, more invasive treatments such as vaginal devices, anal plugs, sphincteroplasty, and neuromodulation are considered. Sacral neuromodulation (SNM), currently the only approved neuromodulation modality in the USA for the treatment of FI, is a safe and effective option for refractory FI [2, 6]. However, it is invasive, expensive, and associated with surgical morbidities [7]. Therefore, percutaneous tibial nerve stimulation (PTNS), an approved therapy for urge urinary incontinence, gained interest as a potential minimally invasive office therapy with lower associated morbidities and cost [8].

Two multi-center randomized trials of PTNS have demonstrated no significant difference in primary ABL outcomes compared with sham treatment, although a significant number of participants improved in each treatment arm [9, 10]. Subsequent post hoc analyses of the CONTrol of Faecal Incontinence using Distal NeuromodulaTion (CONFIDeNT) trial demonstrated significant response to PTNS in a subset of participants without obstructive defecation symptoms[11], suggesting PTNS as a potential treatment for select women. The NeurOmodulatTion for Accidental Bowel Leakage (NOTABLe) trial also found no significant difference in improvement in ABL based on the change in St. Mark’s score after 12 weeks of treatment between those randomized to PTNS vs sham [10]. The objective of this planned secondary analysis was to identify potential subgroups who may experience significant treatment success for PTNS over sham treatment.

## Materials and methods

The study methods and primary results of the NOTABLe trial have been published [10, 12]. The primary study was IRB approved, with a formal Data and Safety Monitoring Board, and was conducted under a single institutional review board approval by the University of Pittsburgh (NCT 03278613). All participants provided written informed consent.

Eligible women 18 years of age or older with at least 3 months of ABL symptoms and a St. Mark’s score of 12 or greater were randomized 2:1 to either PTNS or sham treatment. A 4-week run-in was conducted to exclude those who reported symptom reduction below the inclusion threshold in response to completing bowel diaries and to self-imposed dietary and behavioral measures alone. Baseline measures included participant demographics, clinical characteristics, medical history and condition-specific ABL severity measures prior to run-in. Baseline quality of life measures were collected after run-in, prior to study-treatment initiation. Initial analyses for the primary study outcome of treatment response (i.e., treatment success) were performed using ≥4-point reduction from baseline in St. Mark’s score. Because published minimum important differences are reported to range from 3- to 5-point reductions in St. Mark’s score [13], treatment response success rates were also explored for ≥3 and ≥5 reductions. We further sought to determine whether additional subjective and objective measures provided consistent results. Thus, we examined success definitions using the Patient Global Impression of Improvement (PGI-I) of very much or much better, and using fecal incontinence episodes (FIEs), of ≥50% reduction from baseline as reported on a 14-day Bowel eDiary [14]. All measures were recorded after 12 weekly sessions of therapy.

Baseline demographic, clinical, and symptom characteristics were compared between treatment success and failure for each of the above definitions. Baseline symptom severity was established prior to the 4-week run-in and was defined as baseline St. Mark’s score for the primary treatment responder outcome, baseline patient global symptom control (PGSC) for the PGI-I outcome, and baseline average FIE per week for the FIE success outcome. Diet was assessed by measuring dietary and supplemental fiber intake as well as a 15-item meat/snack questionnaire that assesses intake of dietary fat (scores ranging from 0 to 60 with higher scores indicating greater dietary fat intake) [15]. Additional baseline bowel diary data included number of bowel movements per week, bowel movements with urgency per week, accident-free days per week, leaks per week, and leaks with urgency per week. Each factor was assessed separately for each success outcome (St. Mark’s reduction of ≥3, 4, and 5; PGI-I and ≥50% FIE reduction at 12 weeks).

Descriptive statistics were used to evaluate success rates for each of the above subjective and objective definitions of success. Given differing rates of success by definition, factors associated with success for each definition were explored using bivariate analyses with Chi-squared tests, Student’s *t* tests, and Wilcoxon rank-sum tests as appropriate to identify potential demographic and clinical characteristics associated with treatment effect. Given the substantial improvements observed in the sham treatment group in NOTABLe [10], all treatment responders regardless of treatment allocation (i.e., PTNS or sham) were grouped to identify factors associated with any ABL intervention. Subgroups of participants who received greater benefit from PTNS than sham were identified by evaluating the statistical interaction between participant characteristics and assigned treatment group in logistic regression models. Initial models included clinical site, assigned treatment group (PTNS or sham), and baseline severity, plus clinical, demographic, and symptom severity variables significant in bivariate comparisons at the *p*<0.2 level along with their interactions with treatment group. Backward selection was employed for model generation, with candidate variables removed from the model based on changes to Akaike’s Information Criteria (AIC). Collinearity among the potential model variables was assessed. Adjusted odds ratios (AORs) and 95% confidence intervals (CIs) described the associations between patient characteristics and the success outcomes. A 5% two-sided significance level was used for all statistical testing; no data imputation was performed and no adjustments for multiple testing were made. Analyses were performed using SAS statistical software, version 9.4 (SAS Institute, Inc., Cary, NC, USA).

## Results

Of the 166 women who completed the 4-week run-in phase and were ultimately randomized to treatment, 162 provided some post-baseline data (108 PTNS, 54 sham) and 160 (96%) had sufficient data after 12 weeks of treatment to determine their status based on at least one of the definitions of success. Complete outcome data, inclusive of St. Mark’s score, PGI-I, and bowel diaries were available for 142 (86%) participants. Among the 160 women included in these analyses, mean age at baseline was 64 (±12) years, with 11% Black, 9% Latina, and 80% white self-identified race and ethnicity, and a mean body mass index (BMI) of 29 (±7 kg/m^2^) and baseline St. Mark’s score of 18 (±3).

**Fig. 1:**
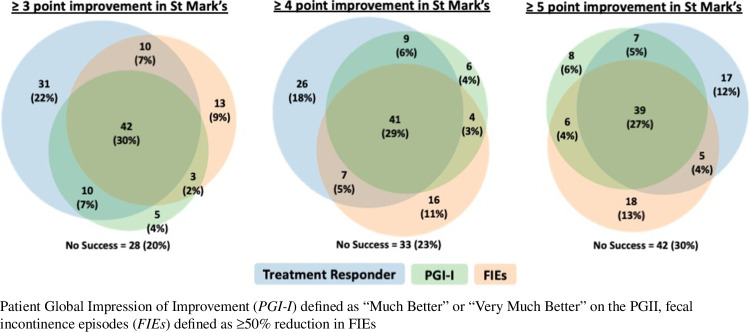
Treatment responder defined by improvement in St Mark’s score from baseline to 12 weeks

The proportion meeting criteria for success was 65% (102 out of 158: 68 PTNS, 34 sham), 57% (90 out of 158: 64 PTNS, 26 sham), and 46% (73 out of 158: 52 PTNS, 21 sham) for the responder outcome (≥3-, 4-, and 5-point reduction from baseline in St. Mark’s score respectively), 43% (68 out of 157: 47 PTNS, 21 sham) for the PGI-I, and 48% (70 out of 145: 51 PTNS, 19 sham) based on the diary variable of ≥50% reduction in FIEs. We also assessed overlaps in success definitions among the 142 women who provided data for all definitions of success. When defining the responder status as ≥4-point change from baseline in St. Mark’s (the NOTABLe primary study outcome), 77% (109 out of 142) met 1 or more success criteria, 43% (61 out of 142) met 2 criteria, and 29% (41 out of 142) met all 3 success criteria. There was no marked change in concordance when the St. Mark’s threshold was changed. Using responder status as ≥3-point reduction in St. Mark’s score, 80% (114 out of 142) met 1 or more success criterion, 46% (65 out of 142) met 2 criteria, and 30% (42 out of 142) met all 3 success criteria. Meanwhile, using responder status as ≥5-point reduction in St. Mark’s score, 70% (100 out of 142) met 1 or more success criteria, 40% (57 out of 142) met 2 criteria, and 27% (39 out of 142) met all 3 success criteria. Figure 1 demonstrates the overlap in various success outcomes within this population using each of the St. Mark’s minimally important difference cutoff points. Of the 83 women with available data for all success definitions and who met criteria for treatment response in the St. Mark’s questionnaire (58% of the total), only 50 (35% of the total) responded that their symptoms were much or very much better. Additionally, there were 41 women (29% of the total) who had success according to St. Mark’s or PGI-I criteria but did not have ≥50% reductions in FIE. The largest discordance was observed among individuals who were classified as treatment responders but did not have a ≥50% reduction in FIE or did not report themselves to be much or very much better on the PGI-I (*n*=26), regardless of the St. Mark’s score cutoff point utilized to define treatment response.

Baseline demographic, clinical, and incontinence severity characteristics of women with vs without success (based on response of ≥4-point reduction in St. Mark’s score, PGI-I, and ≥50% reduction in FIEs) had minimal overlap. Supplemental Tables 1 and 2 provide baseline characteristic comparisons between responders and nonresponders for each definition. Results of the multivariate logistic regression models with evidence of treatment effects are included in Table 1, using the St. Mark’s reduction ≥4 along with PGI-I and ≥50% improvement in FIEs.

**Table 1:** Multivariate logistic regression model predicting treatment success by success definition

Model effect	Model *p* value	Comparison^a^				Odds ratio (95% confidence interval)^b^	Odds ratio *p* value
		Group 1		Group 2			
		Category	*n*/*N* (%)	Category	*n*/*N* (%)		
Responder (≥4-point decrease in St. Mark’s score)							
Site	0.161						
St. Mark’s score (start of run-in)	0.201					1.11 (0.94, 1.30)	0.200
Body mass index (categorical)	0.069	<25 kg/m^2^ with PTNS	22/27 (81)	25–29.9 kg/m^2^ with PTNS	22/36 (61)	4.28 (1.06, 17.26)	0.040*
		<25 kg/m^2^ with PTNS	22/27 (81)	≥30 kg/m^2^ with PTNS	20/40 (50)	6.93 (1.71, 28.11)	0.006*
		25–29.9 kg/m^2^ with PTNS	22/36 (61)	≥30 kg/m^2^ with PTNS	20/40 (50)	1.61 (0.54, 4.83)	0.388
		<25 kg/m^2^ with Sham	5/11 (45)	25–29.9 kg/m^2^ with Sham	11/15 (73)	0.20 (0.02, 1.46)	0.114
		<25 kg/m^2^ with Sham	5/11 (45)	≥30 kg/m^2^ with Sham	10/27 (37)	1.03 (0.16, 6.43)	0.974
		25–29.9 kg/m^2^ with Sham	11/15 (73)	≥30 kg/m^2^ with Sham	10/27 (37)	5.04 (1.05, 24.24)	0.043*
Previous UI surgery	0.094	Yes with PTNS	19/25 (76)	No with PTNS	45/79 (57)	5.01 (1.41, 17.72)	0.012*
		Yes with Sham	7/14 (50)	No with Sham	19/40 (48)	1.11 (0.23, 5.32)	0.889
Treatment	0.031	**PTNS where BMI: <25 kg/m**^**2**^	**22/27 (81)**	**Sham where BMI: <25 kg/m**^**2**^	**5/11 (45)**	**16.61 (2.20, 125.18)**	0.006*
		PTNS where BMI: 25–29.9 kg/m^2^	22/36 (61)	Sham where BMI: 25–29.9 kg/m^2^	11/15 (73)	0.79 (0.15, 3.93)	0.774
		PTNS where BMI: ≥30 kg/m^2^	20/40 (50)	Sham where BMI: >= 30 kg/m^2^	10/27 (37)	2.46 (0.73, 8.32)	0.145
		**PTNS with previous UI surgery**	**19/25 (76)**	**Sham with previous UI surgery**	**7/14 (50)**	**6.75 (1.10, 41.3)**	0.038*
		PTNS without previous UI surgery	45/79 (57)	Sham without previous UI surgery	19/40 (48)	1.50 (0.57, 3.96)	0.407
Interaction of treatment and body mass index (categorical)	0.045						
Interaction of treatment and previous UI surgery	0.140						
**“Much better” or “Very much better” on Patient Global Impression of Improvement**							
Site	0.366						
Patient Global Symptom Control (baseline)	0.734	Control	12/26 (46)	No control	56/130 (43)	0.83 (0.29, 2.38)	0.733
Currently using estrogen	0.074	Yes	13/44 (30)	No	55/113 (49)	0.43 (0.17, 1.09)	0.074
Meat/snack score	0.008	PTNS	n/a	n/a	n/a	1.01 (0.96, 1.06)	0.693
		Sham	n/a	n/a	n/a	1.16 (1.04, 1.30)	0.006*
Education	0.719	Some college or greater with PTNS	27/71 (38)	No college education with PTNS	20/32 (63)	0.47 (0.17, 1.30)	0.146
		Some college or greater with Sham	15/37 (41)	No college education with Sham	6/17 (35)	2.99 (0.58, 15.42)	0.189
Treatment	0.011	PTNS with some college or greater	27/71 (38)	Sham with some college or greater	15/37 (41)	0.76 (0.29, 2.03)	0.594
		PTNS with no college education	20/32 (63)	Sham with no college education	6/17 (35)	4.83 (0.96, 24.18)	0.055
		**PTNS at quartile 1 of meat/snack score**	**n/a**	**Sham at quartile 1 of meat/snack score**	**n/a**	**4.25 (1.25, 14.45)**	0.020*
		PTNS at quartile 2 of meat/snack score	n/a	Sham in at quartile 2 of meat/snack score	n/a	2.38 (0.90, 6.28)	0.079
		PTNS at quartile 3 of meat/snack score	n/a	Sham at quartile 3 of meat/snack score	n/a	0.99 (0.37, 2.68)	0.997
Interaction of meat/snack score and treatment	0.018						
Interaction of treatment and education	0.059						
**Objective success: ≥ 50% decrease in FIEs/week**							
Site	0.032						
FIE per week (baseline)	0.146	PTNS	n/a	n/a	n/a	0.99 (0.92, 1.07)	0.864
		Sham	n/a	n/a	n/a	0.86 (0.72, 1.04)	0.127
Currently using estrogen	0.020	Yes	14/42 (33)	No	56/103 (54)	0.26 (0.08, 0.81)	0.019*
Hysterectomy	0.043	Yes	26/68 (38)	No	44/77 (57)	0.34 (0.12, 0.97)	0.042*
Pain/discomfort 6 months or longer	0.004	Yes	40/70 (57)	No	30/75 (40)	4.10 (1.57, 10.66)	0.003*
Taking fiber supplements	0.002	Yes with PTNS	22/46 (48)	No with PTNS	28/49 (57)	0.64 (0.22, 1.85)	0.411
		Yes with Sham	2/15 (13)	No with Sham	16/30 (53)	0.03 (0.00, 0.31)	0.002*
Previous POP surgery	0.148	Yes	15/39 (38)	No	55/106 (52)	2.58 (0.71, 9.38)	0.147
Treatment	0.731	**PTNS with fiber supplementation**	**22/46 (48)**	**Sham with fiber supplementation**	**2/15 (13)**	**15.20 (1.95, 118.35)**	0.009*
		PTNS without fiber supplementation	28/49 (57)	Sham without fiber supplementation	16/30 (53)	0.87 (0.27, 2.80)	0.825
		PTNS at quartile 1 of leaks per week	n/a	Sham at quartile 1 of leaks per week	n/a	2.08 (0.65, 6.64)	0.215
		PTNS at quartile 2 of leaks per week	n/a	Sham at quartile 2 of leaks per week	n/a	2.91 (0.94, 8.96)	0.062
		**PTNS at quartile 3 of leaks per week**	**n/a**	**Sham at quartile 3 of leaks per week**	**n/a**	**5.34 (1.19, 23.96)**	0.028*
Interaction of FIE per week (baseline) and treatment	0.166						
Interaction of treatment and fiber supplements	0.015						

*BMI* body mass index, *FIE* fecal incontinence episode, *ABL* accidental bowel leakage, *UI* urinary incontinence, *POP*, pelvic organ prolapse, *PTNS* percutaneous tibial nerve stimulation, *SD* standard deviation

*Indicates a statistically significant effect in the model

^a^Comparisons provided for all categorical terms and interactions retained within the model. Comparator groups (i.e., group 1 and group 2) are only provided for categorical variables where applicable. In those interactions containing categorical and continuous variables, the provided odds ratios are calculated for one unit change in the continuous value and comparators values are designated with ‘n/a’

^b^Odds ratios calculated from the backward-selected model and are adjusted for the selected risk factors. Odds ratios for risk factors included in interactions were calculated within each level of the other interaction variable

For the outcome of responders based on St. Mark’s score, the only statistically significant interaction effects between treatment groups were noted for BMI and previous urinary incontinence (UI) surgery. In these interactions, women with normal or underweight BMI (<25 kg/m^2^) or with previous UI surgery were more likely to be treatment responders when receiving PTNS than sham (BMI: AOR 16.61 (95% CI 2.20, 125.18), *p*<0.01; previous UI surgery: AOR 6.76 (95% CI 1.10 41.30), *p*<0.05).

For PGI-I, interactions were observed between treatment group and both meat/snack score and education. Women reporting lowest fat intake (lowest tertile on the meat/snack scores) who were randomized to PTNS were more likely than the sham group to express an impression of improvement (AOR 4.25 (95% CI 1.25, 14.45), *p*<0.05). Further examination of the interaction with education did not reveal greater benefit from PTNS versus sham for any education subgroup.

For objective success of ≥50% improvement in FIEs on the 14-day bowel diary, there were interaction effects between treatment group and both fiber supplementation and baseline FIE frequency. Although overall success was lower in those women on fiber supplementation, those on fiber supplements who received PTNS were more likely to experience objective success than those who received sham (AOR 15.20 [95% CI 1.95, 118.35], *p*<0.01); however, this was driven primarily by a lack of response in the sham group. Additionally, participants with higher baseline FIE frequency were more likely to experience objective success in the PTNS group than in the sham group (AOR 5.34 [95% CI 1.19, 23.96], *p*<0.05).

Given the observed associations with dietary factors of fiber and fat intake, a supplemental analysis was conducted that examined the relationship between baseline fiber supplement use and stool consistency, as measured by the Bristol Stool Scale. No association between fiber supplement use and the Bristol Stool Scale score was observed (data not shown).

There were no significant treatment effects for PTNS for success definitions using St. Mark’s reduction of ≥3 or ≥5 (Supplemental Table 3).

## Discussion

The NOTABLe trial is the second multicenter, randomized, masked trial of PTNS for treatment of ABL to report no benefit over sham stimulation [9, 10]. In this planned secondary analysis, we aimed to determine whether patient characteristics were associated with PTNS treatment benefit in order to potentially identify a subgroup of patients who may benefit from peripheral neuromodulation. No consistent predictors of response to treatment were identified in this analysis across multiple definitions of success using both subjective and objective criteria. Furthermore, in reviewing these data, we found that success rates varied widely according to the success definition used, with only 29% meeting success according to all three of the analyzed criteria based on St. Mark’s ≥4-point improvement, PGI-I, and reductions in FIE, whereas 77% of the study population met at least one of the success definitions.

This heterogeneity in outcome status is notable, as it highlights the importance of the definition utilized when assessing ABL treatment efficacy and suggests potential variability in standardized research trials compared with common clinical intervention. Indeed, prior research of pelvic floor dysfunction has revealed discordance in the patient assessment of treatment success compared with objective measures with respect to surgical treatment of pelvic organ prolapse [16, 17]. However, unlike prior studies that showed a strong correlation of subjective impressions of patient improvement, even when discordant from objective anatomical measures, our data suggest that, among women with refractory ABL, there remains substantial discordance in patient assessment of treatment success, with less than one third of women reporting subjective success without evidence of objective success. Given that objective outcomes are typically required for permanent implantation of a sacral neuromodulation device, these findings further highlight the potential weakness of our existing disease severity measures. Moreover, the optimal cutoff point for the St. Mark’s score minimally important differences is not universally accepted and ranges from 3 to 5 [13]. In this cohort of women, the variation in cutoff point definition resulted in a variation in success rates of nearly 20%. Additionally, using the 3- and 5-point thresholds revealed no consistent treatment effects between PTNS and sham and did not improve concordance in the success definitions. Based on these findings, we suggest further investigation into the optimal definitions of success and failure for ABL intervention trials.

With respect to potential differential efficacy of PTNS vs sham, although there were isolated risk factors that met the significance criteria in our analyses, they were not consistent across our outcome measures or differential response thresholds. As such, our study failed to identify clinically meaningful differences in the application of PTNS to any subgroup of women with ABL. Notably, however, this analysis was exploratory in nature, did not include qualitative assessments and the study was not designed or powered to find differences in response between subgroups. Additionally, this study did not perform sophisticated characterization of anal sphincter defects, rectal tone, and compliance or pudendal neuropathy. Though not part of the NOTABLe study design, high-resolution anorectal manometry with rectal sensory and compliance testing may have helped to characterize individuals with affected rectal tone and compliance who may have differing responses to treatment. Additionally, NOTABLe did not ascertain through questionnaire or structured interview baseline obstructive defecation (OD) symptoms of difficulty with rectal evacuation, excessive straining, requirement for regular digitation, or a sensation of incomplete emptying. As such, we cannot support or refute the negative association between OD and PTNS treatment outcomes identified in a post-hoc subgroup analysis of the CONFIDeNT trial [11].

Our study findings are strengthened by the use of a large number of participants with uniform, validated data collection from a multicenter, randomized trial. However, given the inclusion criteria applied to the NOTABLe trial, the results are not generalizable to men or to individuals with less-severe ABL undergoing behavioral or pharmacological treatment. Fecal incontinence is known to have a significant impact on patient wellbeing and quality of life, but those influences are multifactorial and individualized [18]. Given the differential outcome experiences, as well as the heterogeneity in success outcomes demonstrated here and in other intervention studies [19], our findings further emphasize the importance of careful counseling to assess patient bother, as well as treatment goals and expectations. Further evaluation of existing novel outcome measures, including the Accident Bowel Leakage Evaluation [20] instrument as a potential outcome measure of treatment response, may help in the quest for the appropriate outcome assessment for ABL treatments.

## Conclusion

Our data reinforce prior findings and fail to find consistent predictors of response to PTNS for the treatment of women with refractory ABL. However, these data do suggest that there remains substantial discordance in outcomes across the various current measures of fecal incontinence. Further assessment of optimal outcomes for ABL treatment is needed. These findings may subsequently benefit future investigations of interventions for ABL.

## Supplementary information

ESM 1 (DOCX 75 kb)

ESM 2 (DOCX 54 kb)

ESM 3 (DOCX 42 kb)
