# Supplementary material for: Characteristics associated with subjective and objective measures of treatment success in women undergoing percutaneous tibial nerve stimulation vs sham for accidental bowel leakage
Source: Int Urogynecol J. 2023 Jan 27;34(8):1715–23. doi: 10.1007/s00192-022-05431-y (PMC10372194; doi:10.1007/s00192-022-05431-y)
Supplement: Supplementary file 1 — (DOCX 75 kb) [file 192_2022_5431_MOESM1_ESM.docx]

**Supplemental Table 1. Characteristics of Women with Success vs Failure by Success Outcome Definition.**

|  | | | ***Responder Status (>= 4-point decrease in St. Mark's Score)*** | | | ***Patient Global Impression of Improvement*** | | | ***50% Improvement in FIEs*** | | | |
| --- | --- | --- | --- | --- | --- | --- | --- | --- | --- | --- | --- | --- |
| ***Characteristic*** | ***Category*** | ***Total (N=160)*** | ***Yes (N=90)*** | ***No (N=68)*** | ***P-value ^a^*** | ***Yes (N=68)*** | ***No (N=89)*** | ***P-value ^a^*** | ***Yes (N=70)*** | ***No (N=75)*** | ***P-value ^a^*** | |
| Randomized Treatment, n/N (%) | PTNS | 106/160 (66.3) | 64/90 (71.1) | 40/68 (58.8) | 0.108* | 47/68 (69.1) | 56/89 (62.9) | 0.419 | 51/70 (72.9) | 46/75 (61.3) | 0.142* | |
|  | Sham | 54/160 (33.8) | 26/90 (28.9) | 28/68 (41.2) |  | 21/68 (30.9) | 33/89 (37.1) |  | 19/70 (27.1) | 29/75 (38.7) |  | |
|  | | | | | | | | | | | | |
| Age, mean (SD) [min, max], y |  | 63.6 (11.5) [29.0, 90.0] | 64.4 (11.6) [29.0, 85.0] | 62.8 (11.4) [34.0, 90.0] | 0.380 | 62.6 (11.7) [29.0, 84.0] | 64.8 (11.2) [33.0, 90.0] | 0.230 | 62.6 (12.9) [29.0, 90.0] | 64.9 (10.2) [37.0, 85.0] | 0.222 | |
|  | | | | | | | | | | | | |
| Race, n/N (%) ^b^ | American Indian or Alaska Native | 2/160 (1.3) | 2/90 (2.2) | 0/68 (0.0) | 0.999 | 0/68 (0.0) | 2/89 (2.2) | 0.758 | 0/70 (0.0) | 2/75 (2.7) | 1.000 | |
|  | Asian | 2/160 (1.3) | 1/90 (1.1) | 1/68 (1.5) |  | 1/68 (1.5) | 1/89 (1.1) |  | 2/70 (2.9) | 0/75 (0.0) |  | |
|  | Black or African American | 19/160 (11.9) | 11/90 (12.2) | 7/68 (10.3) |  | 10/68 (14.7) | 8/89 (9.0) |  | 9/70 (12.9) | 9/75 (12.0) |  | |
|  | Native Hawaiian or Other Pacific Islander | 2/160 (1.3) | 0/90 (0.0) | 2/68 (2.9) |  | 0/68 (0.0) | 1/89 (1.1) |  | 0/70 (0.0) | 2/75 (2.7) |  | |
|  | White | 128/160 (80.0) | 72/90 (80.0) | 55/68 (80.9) |  | 52/68 (76.5) | 75/89 (84.3) |  | 57/70 (81.4) | 59/75 (78.7) |  | |
|  | More than one race | 3/160 (1.9) | 2/90 (2.2) | 1/68 (1.5) |  | 2/68 (2.9) | 1/89 (1.1) |  | 1/70 (1.4) | 2/75 (2.7) |  | |
|  | Unknown/Not Reported | 4/160 (2.5) | 2/90 (2.2) | 2/68 (2.9) |  | 3/68 (4.4) | 1/89 (1.1) |  | 1/70 (1.4) | 1/75 (1.3) |  | |
|  | | | | | | | | | | | | |
| Ethnicity, n/N (%) | Hispanic/Latina | 15/160 (9.4) | 9/90 (10.0) | 5/68 (7.4) | 0.836 | 10/68 (14.7) | 4/89 (4.5) | 0.102* | 7/70 (10.0) | 6/75 (8.0) | 0.904 | |
|  | Not Hispanic/Latina | 144/160 (90.0) | 80/90 (88.9) | 63/68 (92.6) |  | 57/68 (83.8) | 85/89 (95.5) |  | 62/70 (88.6) | 69/75 (92.0) |  | |
|  | Unknown/Not Reported | 1/160 (0.6) | 1/90 (1.1) | 0/68 (0.0) |  | 1/68 (1.5) | 0/89 (0.0) |  | 1/70 (1.4) | 0/75 (0.0) |  | |
|  | | | | | | | | | | | | |
| Primary language, n/N (%) | English | 155/160 (96.9) | 87/90 (96.7) | 66/68 (97.1) | 0.889 | 65/68 (95.6) | 87/89 (97.8) | 0.452 | 69/70 (98.6) | 72/75 (96.0) | 0.365 | |
|  | Spanish | 5/160 (3.1) | 3/90 (3.3) | 2/68 (2.9) |  | 3/68 (4.4) | 2/89 (2.2) |  | 1/70 (1.4) | 3/75 (4.0) |  | |
|  | | | | | | | | | | | | |
| Education, n/N (%) | Some college or greater | 111/160 (69.4) | 66/90 (73.3) | 43/68 (63.2) | 0.176* | 42/68 (61.8) | 66/89 (74.2) | 0.098* | 49/70 (70.0) | 53/75 (70.7) | 0.930 | |
|  | No college education | 49/160 (30.6) | 24/90 (26.7) | 25/68 (36.8) |  | 26/68 (38.2) | 23/89 (25.8) |  | 21/70 (30.0) | 22/75 (29.3) |  | |
|  | | | | | | | | | | | | |
| Insurance status, n/N (%) | Private/HMO | 57/160 (35.6) | 33/90 (36.7) | 24/68 (35.3) | 0.766 | 19/68 (27.9) | 37/89 (41.6) | 0.035* | 21/70 (30.0) | 31/75 (41.3) | 0.126* | |
|  | Medicare/Medicaid | 44/160 (27.5) | 26/90 (28.9) | 17/68 (25.0) |  | 23/68 (33.8) | 20/89 (22.5) |  | 25/70 (35.7) | 15/75 (20.0) |  | |
|  | Both Private and Medicare/Medicaid | 41/160 (25.6) | 20/90 (22.2) | 20/68 (29.4) |  | 14/68 (20.6) | 26/89 (29.2) |  | 15/70 (21.4) | 22/75 (29.3) |  | |
|  | Other/None | 18/160 (11.3) | 11/90 (12.2) | 7/68 (10.3) |  | 12/68 (17.6) | 6/89 (6.7) |  | 9/70 (12.9) | 7/75 (9.3) |  | |
|  | | | | | | | | | | | | |
| Body Mass Index, mean (SD) [min, max] |  | 29.4 (6.7) [18.0, 55.0] | 28.3 (5.7) [19.0, 44.0] | 31.0 (7.5) [18.0, 55.0] | 0.016* | 30.2 (6.8) [20.0, 55.0] | 28.9 (6.5) [18.0, 55.0] | 0.215 | 29.0 (7.1) [20.0, 55.0] | 28.7 (5.2) [18.0, 42.0] | 0.741 | |
| Body Mass Index, n/N (%) | <25 kg/m2 | 39/158 (24.7) | 27/90 (30.0) | 11/66 (16.7) | 0.017* | 14/67 (20.9) | 23/88 (26.1) | 0.582 | 22/70 (31.4) | 16/74 (21.6) | 0.413 | |
|  | 25 - 29.9 kg/m2 | 51/158 (32.3) | 33/90 (36.7) | 18/66 (27.3) |  | 21/67 (31.3) | 30/88 (34.1) |  | 22/70 (31.4) | 27/74 (36.5) |  | |
|  | >= 30 kg/m2 | 68/158 (43.0) | 30/90 (33.3) | 37/66 (56.1) |  | 32/67 (47.8) | 35/88 (39.8) |  | 26/70 (37.1) | 31/74 (41.9) |  | |
|  | | | | | | | | | | | | |
| Anal sphincter squeeze, n/N (%) |  | 141/158 (89.2) | 81/90 (90.0) | 58/66 (87.9) | 0.675 | 61/67 (91.0) | 78/88 (88.6) | 0.626 | 61/70 (87.1) | 68/74 (91.9) | 0.355 | |
|  | | | | | | | | | | | | |
| Any vaginal deliveries, n/N (%) |  | 141/160 (88.1) | 78/90 (86.7) | 61/68 (89.7) | 0.562 | 58/68 (85.3) | 80/89 (89.9) | 0.384 | 58/70 (82.9) | 68/75 (90.7) | 0.169* | |
| Any cesarean deliveries, n/N (%) |  | 17/160 (10.6) | 9/90 (10.0) | 8/68 (11.8) | 0.723 | 9/68 (13.2) | 7/89 (7.9) | 0.275 | 7/70 (10.0) | 9/75 (12.0) | 0.701 | |
|  | | | | | | | | | | | | |
| Menopausal status, n/N (%) | Pre-menopausal | 14/160 (8.8) | 8/90 (8.9) | 6/68 (8.8) | 0.996 | 7/68 (10.3) | 7/89 (7.9) | 0.793 | 9/70 (12.9) | 5/75 (6.7) | 0.396 | |
|  | Post-menopausal | 137/160 (85.6) | 77/90 (85.6) | 58/68 (85.3) |  | 57/68 (83.8) | 78/89 (87.6) |  | 56/70 (80.0) | 66/75 (88.0) |  | |
|  | Not sure | 9/160 (5.6) | 5/90 (5.6) | 4/68 (5.9) |  | 4/68 (5.9) | 4/89 (4.5) |  | 5/70 (7.1) | 4/75 (5.3) |  | |
|  | | | | | | | | | | | | |
| Currently using estrogen, n/N (%) |  | 46/160 (28.8) | 25/90 (27.8) | 19/68 (27.9) | 0.982 | 13/68 (19.1) | 31/89 (34.8) | 0.032* | 14/70 (20.0) | 28/75 (37.3) | 0.023* | |
|  | | | | | | | | | | | | |
| Current smoker, n/N (%) |  | 13/160 (8.1) | 7/90 (7.8) | 6/68 (8.8) | 0.813 | 8/68 (11.8) | 5/89 (5.6) | 0.175* | 7/70 (10.0) | 4/75 (5.3) | 0.296 | |
|  | | | | | | | | | | | | |
| Urgency Urinary Incontinence, n/N (%) |  | 115/157 (73.2) | 65/90 (72.2) | 48/65 (73.8) | 0.823 | 50/68 (73.5) | 62/86 (72.1) | 0.843 | 49/70 (70.0) | 54/72 (75.0) | 0.506 | |
|  | | | | | | | | | | | | |
| Previous ABL surgery, n/N (%) |  | 8/160 (5.0) | 7/90 (7.8) | 1/68 (1.5) | 0.109* | 3/68 (4.4) | 5/89 (5.6) | 0.734 | 5/70 (7.1) | 3/75 (4.0) | 0.414 | |
| Previous anal/rectal surgery, n/N (%) |  | 27/160 (16.9) | 17/90 (18.9) | 10/68 (14.7) | 0.490 | 13/68 (19.1) | 14/89 (15.7) | 0.578 | 12/70 (17.1) | 12/75 (16.0) | 0.853 | |
| Previous UI surgery, n/N (%) |  | 40/160 (25.0) | 26/90 (28.9) | 13/68 (19.1) | 0.161* | 19/68 (27.9) | 20/89 (22.5) | 0.433 | 16/70 (22.9) | 21/75 (28.0) | 0.478 | |
| Previous POP surgery, n/N (%) |  | 40/160 (25.0) | 25/90 (27.8) | 14/68 (20.6) | 0.301 | 15/68 (22.1) | 24/89 (27.0) | 0.481 | 15/70 (21.4) | 24/75 (32.0) | 0.154* | |
| Hysterectomy, n/N (%) |  | 75/160 (46.9) | 42/90 (46.7) | 33/68 (48.5) | 0.816 | 33/68 (48.5) | 41/89 (46.1) | 0.759 | 26/70 (37.1) | 42/75 (56.0) | 0.024* | |
|  | | | | | | | | | | | | |
| Taking fiber supplements, n/N (%) |  | 65/155 (41.9) | 37/87 (42.5) | 28/66 (42.4) | 0.990 | 27/66 (40.9) | 37/86 (43.0) | 0.794 | 24/68 (35.3) | 37/72 (51.4) | 0.056* | |
| Dietary fiber intake, mean (SD) [min, max], g |  | 13.9 (4.3) [4.9, 30.0] | 13.9 (4.3) [4.9, 30.0] | 13.8 (4.3) [5.6, 27.1] | 0.944 | 14.5 (4.4) [4.9, 30.0] | 13.4 (4.1) [5.6, 27.1] | 0.129* | 14.1 (4.3) [4.9, 30.0] | 14.0 (4.4) [5.6, 25.6] | 0.889 | |
| Meat/Snack Screener Score, mean (SD) [min, max] |  | 18.3 (8.8) [0.0, 52.0] | 18.3 (9.3) [0.0, 52.0] | 18.4 (8.1) [2.0, 43.0] | 0.960 | 20.0 (10.2) [2.0, 52.0] | 17.2 (7.3) [0.0, 41.0] | 0.057* | 18.6 (9.5) [2.0, 52.0] | 17.7 (7.7) [0.0, 41.0] | 0.519 | |
|  | | | | | | | | | | | | |
| Bristol Stool Type, n/N (%) | Type 2 - Sausage-shaped but lumpy | 12/160 (7.5) | 5/90 (5.6) | 6/68 (8.8) | 0.506 | 6/68 (8.8) | 5/89 (5.6) | 0.656 | 5/70 (7.1) | 6/75 (8.0) | 0.282 | |
|  | Type 3 - Like a sausage but with cracks on its surface | 22/160 (13.8) | 14/90 (15.6) | 7/68 (10.3) |  | 8/68 (11.8) | 13/89 (14.6) |  | 12/70 (17.1) | 10/75 (13.3) |  | |
|  | Type 4 - Like a sausage or snake, smooth and soft | 42/160 (26.3) | 22/90 (24.4) | 20/68 (29.4) |  | 15/68 (22.1) | 26/89 (29.2) |  | 13/70 (18.6) | 26/75 (34.7) |  | |
|  | Type 5 - Soft blobs with clear-cut edges | 33/160 (20.6) | 22/90 (24.4) | 11/68 (16.2) |  | 17/68 (25.0) | 16/89 (18.0) |  | 17/70 (24.3) | 13/75 (17.3) |  | |
|  | Type 6 - Fluffy pieces with ragged edges, a mushy stool | 51/160 (31.9) | 27/90 (30.0) | 24/68 (35.3) |  | 22/68 (32.4) | 29/89 (32.6) |  | 23/70 (32.9) | 20/75 (26.7) |  | |
|  | | | | | | | | | | | | |
| Pain/discomfort in abdomen in last 3 months, n/N (%) | Less than once per week | 99/160 (61.9) | 55/90 (61.1) | 43/68 (63.2) | 0.786 | 42/68 (61.8) | 55/89 (61.8) | 0.997 | 39/70 (55.7) | 51/75 (68.0) | 0.129* | |
|  | At least once per week | 61/160 (38.1) | 35/90 (38.9) | 25/68 (36.8) |  | 26/68 (38.2) | 34/89 (38.2) |  | 31/70 (44.3) | 24/75 (32.0) |  | |
| Pain/discomfort 6 months or longer, n/N (%) |  | 79/160 (49.4) | 42/90 (46.7) | 36/68 (52.9) | 0.435 | 33/68 (48.5) | 45/89 (50.6) | 0.801 | 40/70 (57.1) | 30/75 (40.0) | 0.040* | |
| Diagnosed with IBS, n/N (%) |  | 35/160 (21.9) | 16/90 (17.8) | 18/68 (26.5) | 0.279 | 13/68 (19.1) | 21/89 (23.6) | 0.793 | 15/70 (21.4) | 15/75 (20.0) | 0.943 | |
| Frequency of loose/mushy/watery stools in last 3 months, n/N (%) | Never or rare | 55/160 (34.4) | 31/90 (34.4) | 23/68 (33.8) | 0.860 | 18/68 (26.5) | 35/89 (39.3) | 0.244 | 18/70 (25.7) | 33/75 (44.0) | 0.065* | |
|  | Sometimes | 33/160 (20.6) | 20/90 (22.2) | 13/68 (19.1) |  | 16/68 (23.5) | 17/89 (19.1) |  | 16/70 (22.9) | 15/75 (20.0) |  | |
|  | Often/most of the time/always | 72/160 (45.0) | 39/90 (43.3) | 32/68 (47.1) |  | 34/68 (50.0) | 37/89 (41.6) |  | 36/70 (51.4) | 27/75 (36.0) |  | |
|  | | | | | | | | | | | | |
| St. Mark's Score (start of run-in), mean (SD) [min, max] |  | 17.9 (2.6) [12.0, 24.0] | 18.2 (2.5) [12.0, 24.0] | 17.6 (2.7) [12.0, 23.0] | 0.146* | 18.5 (2.6) [12.0, 24.0] | 17.5 (2.4) [12.0, 22.0] | 0.019* | 18.2 (2.6) [12.0, 24.0] | 17.5 (2.5) [12.0, 23.0] | 0.118* | |
|  | | | | | | | | | | | | |
| Bowel movements per week (start of run-in), mean (SD) [min, max], No. |  | 12.7 (8.1) [0.0, 45.5] | 13.2 (8.7) [0.0, 45.5] | 12.0 (7.4) [1.0, 38.0] | 0.381 | 12.3 (7.5) [0.0, 43.8] | 13.1 (8.8) [0.0, 45.5] | 0.579 | 13.0 (9.2) [0.0, 45.5] | 12.5 (7.4) [0.0, 38.0] | 0.746 | |
| Bowel movements with urgency per week (start of run-in), mean (SD) [min, max], No. |  | 6.0 (5.4) [0.0, 34.0] | 5.9 (5.2) [0.0, 26.5] | 6.0 (5.7) [0.0, 34.0] | 0.919 | 5.7 (4.5) [0.0, 21.9] | 6.2 (6.0) [0.0, 34.0] | 0.578 | 5.5 (4.8) [0.0, 21.9] | 6.3 (5.9) [0.0, 34.0] | 0.388 | |
| Accident-free days per week (start of run-in), mean (SD) [min, max], No. |  | 3.0 (2.0) [0.0, 7.0] | 3.0 (2.1) [0.0, 7.0] | 3.1 (1.9) [0.0, 7.0] | 0.643 | 3.1 (2.1) [0.0, 7.0] | 2.9 (2.0) [0.0, 7.0] | 0.606 | 3.2 (2.1) [0.0, 7.0] | 3.0 (2.0) [0.0, 7.0] | 0.580 | |
| Leaks per week (start of run-in), mean (SD) [min, max], No. |  | 8.1 (7.8) [0.0, 52.0] | 8.1 (7.2) [0.0, 36.5] | 8.0 (8.6) [0.0, 52.0] | 0.900 | 7.4 (6.0) [0.0, 26.0] | 8.7 (9.0) [0.0, 52.0] | 0.312 | 7.4 (7.6) [0.0, 39.0] | 7.9 (6.3) [0.0, 27.0] | 0.625 | |
| Leaks with urgency per week (start of run-in), mean (SD) [min, max], No. |  | 3.4 (4.3) [0.0, 35.0] | 3.2 (3.9) [0.0, 25.0] | 3.5 (4.9) [0.0, 35.0] | 0.655 | 3.2 (3.0) [0.0, 14.0] | 3.5 (5.2) [0.0, 35.0] | 0.677 | 2.7 (2.5) [0.0, 10.0] | 3.3 (3.6) [0.0, 16.0] | 0.223 | |
|  | | | | | | | | | | | | |
| Patient Global Symptom Control, n/N (%) |  | 28/159 (17.6) | 16/89 (18.0) | 11/68 (16.2) | 0.767 | 12/68 (17.6) | 14/88 (15.9) | 0.773 | 13/70 (18.6) | 11/74 (14.9) | 0.552 | |
|  | | | | | | | | | | | | |
|  | | | ***Responder Status (>= 4-point decrease in St. Mark's Score)*** | | | ***Patient Global Impression of Improvement*** | | | ***50% Improvement in FIEs*** | | |  |
| ***Characteristic*** | ***Category*** | ***Total (N=160)*** | ***Yes (N=90)*** | ***No (N=68)*** | ***P-value ^a^*** | ***Yes (N=68)*** | ***No (N=89)*** | ***P-value ^a^*** | ***Yes (N=70)*** | ***No (N=75)*** | ***P-value ^a^*** |  |
| Randomized Treatment, n/N (%) | PTNS | 106/160 (66.3) | 64/90 (71.1) | 40/68 (58.8) | 0.108* | 47/68 (69.1) | 56/89 (62.9) | 0.419 | 51/70 (72.9) | 46/75 (61.3) | 0.142* |  |
|  | Sham | 54/160 (33.8) | 26/90 (28.9) | 28/68 (41.2) |  | 21/68 (30.9) | 33/89 (37.1) |  | 19/70 (27.1) | 29/75 (38.7) |  |  |
|  | | | | | | | | | | | |  |
| Age, mean (SD) [min, max], y |  | 63.6 (11.5) [29.0, 90.0] | 64.4 (11.6) [29.0, 85.0] | 62.8 (11.4) [34.0, 90.0] | 0.380 | 62.6 (11.7) [29.0, 84.0] | 64.8 (11.2) [33.0, 90.0] | 0.230 | 62.6 (12.9) [29.0, 90.0] | 64.9 (10.2) [37.0, 85.0] | 0.222 |  |
|  | | | | | | | | | | | |  |
| Race, n/N (%) ^b^ | American Indian or Alaska Native | 2/160 (1.3) | 2/90 (2.2) | 0/68 (0.0) | 0.999 | 0/68 (0.0) | 2/89 (2.2) | 0.758 | 0/70 (0.0) | 2/75 (2.7) | 1.000 |  |
|  | Asian | 2/160 (1.3) | 1/90 (1.1) | 1/68 (1.5) |  | 1/68 (1.5) | 1/89 (1.1) |  | 2/70 (2.9) | 0/75 (0.0) |  |  |
|  | Black or African American | 19/160 (11.9) | 11/90 (12.2) | 7/68 (10.3) |  | 10/68 (14.7) | 8/89 (9.0) |  | 9/70 (12.9) | 9/75 (12.0) |  |  |
|  | Native Hawaiian or Other Pacific Islander | 2/160 (1.3) | 0/90 (0.0) | 2/68 (2.9) |  | 0/68 (0.0) | 1/89 (1.1) |  | 0/70 (0.0) | 2/75 (2.7) |  |  |
|  | White | 128/160 (80.0) | 72/90 (80.0) | 55/68 (80.9) |  | 52/68 (76.5) | 75/89 (84.3) |  | 57/70 (81.4) | 59/75 (78.7) |  |  |
|  | More than one race | 3/160 (1.9) | 2/90 (2.2) | 1/68 (1.5) |  | 2/68 (2.9) | 1/89 (1.1) |  | 1/70 (1.4) | 2/75 (2.7) |  |  |
|  | Unknown/Not Reported | 4/160 (2.5) | 2/90 (2.2) | 2/68 (2.9) |  | 3/68 (4.4) | 1/89 (1.1) |  | 1/70 (1.4) | 1/75 (1.3) |  |  |
|  | | | | | | | | | | | |  |
| Ethnicity, n/N (%) | Hispanic/Latina | 15/160 (9.4) | 9/90 (10.0) | 5/68 (7.4) | 0.836 | 10/68 (14.7) | 4/89 (4.5) | 0.102* | 7/70 (10.0) | 6/75 (8.0) | 0.904 |  |
|  | Not Hispanic/Latina | 144/160 (90.0) | 80/90 (88.9) | 63/68 (92.6) |  | 57/68 (83.8) | 85/89 (95.5) |  | 62/70 (88.6) | 69/75 (92.0) |  |  |
|  | Unknown/Not Reported | 1/160 (0.6) | 1/90 (1.1) | 0/68 (0.0) |  | 1/68 (1.5) | 0/89 (0.0) |  | 1/70 (1.4) | 0/75 (0.0) |  |  |
|  | | | | | | | | | | | |  |
| Primary language, n/N (%) | English | 155/160 (96.9) | 87/90 (96.7) | 66/68 (97.1) | 0.889 | 65/68 (95.6) | 87/89 (97.8) | 0.452 | 69/70 (98.6) | 72/75 (96.0) | 0.365 |  |
|  | Spanish | 5/160 (3.1) | 3/90 (3.3) | 2/68 (2.9) |  | 3/68 (4.4) | 2/89 (2.2) |  | 1/70 (1.4) | 3/75 (4.0) |  |  |
|  | | | | | | | | | | | |  |
| Education, n/N (%) | Some college or greater | 111/160 (69.4) | 66/90 (73.3) | 43/68 (63.2) | 0.176* | 42/68 (61.8) | 66/89 (74.2) | 0.098* | 49/70 (70.0) | 53/75 (70.7) | 0.930 |  |
|  | No college education | 49/160 (30.6) | 24/90 (26.7) | 25/68 (36.8) |  | 26/68 (38.2) | 23/89 (25.8) |  | 21/70 (30.0) | 22/75 (29.3) |  |  |
|  | | | | | | | | | | | |  |
| Insurance status, n/N (%) | Private/HMO | 57/160 (35.6) | 33/90 (36.7) | 24/68 (35.3) | 0.766 | 19/68 (27.9) | 37/89 (41.6) | 0.035* | 21/70 (30.0) | 31/75 (41.3) | 0.126* |  |
|  | Medicare/Medicaid | 44/160 (27.5) | 26/90 (28.9) | 17/68 (25.0) |  | 23/68 (33.8) | 20/89 (22.5) |  | 25/70 (35.7) | 15/75 (20.0) |  |  |
|  | Both Private and Medicare/Medicaid | 41/160 (25.6) | 20/90 (22.2) | 20/68 (29.4) |  | 14/68 (20.6) | 26/89 (29.2) |  | 15/70 (21.4) | 22/75 (29.3) |  |  |
|  | Other/None | 18/160 (11.3) | 11/90 (12.2) | 7/68 (10.3) |  | 12/68 (17.6) | 6/89 (6.7) |  | 9/70 (12.9) | 7/75 (9.3) |  |  |
|  | | | | | | | | | | | |  |
| Body Mass Index, mean (SD) [min, max] |  | 29.4 (6.7) [18.0, 55.0] | 28.3 (5.7) [19.0, 44.0] | 31.0 (7.5) [18.0, 55.0] | 0.016* | 30.2 (6.8) [20.0, 55.0] | 28.9 (6.5) [18.0, 55.0] | 0.215 | 29.0 (7.1) [20.0, 55.0] | 28.7 (5.2) [18.0, 42.0] | 0.741 |  |
| Body Mass Index, n/N (%) | <25 kg/m2 | 39/158 (24.7) | 27/90 (30.0) | 11/66 (16.7) | 0.017* | 14/67 (20.9) | 23/88 (26.1) | 0.582 | 22/70 (31.4) | 16/74 (21.6) | 0.413 |  |
|  | 25 - 29.9 kg/m2 | 51/158 (32.3) | 33/90 (36.7) | 18/66 (27.3) |  | 21/67 (31.3) | 30/88 (34.1) |  | 22/70 (31.4) | 27/74 (36.5) |  |  |
|  | >= 30 kg/m2 | 68/158 (43.0) | 30/90 (33.3) | 37/66 (56.1) |  | 32/67 (47.8) | 35/88 (39.8) |  | 26/70 (37.1) | 31/74 (41.9) |  |  |
|  | | | | | | | | | | | |  |
| Anal sphincter squeeze, n/N (%) |  | 141/158 (89.2) | 81/90 (90.0) | 58/66 (87.9) | 0.675 | 61/67 (91.0) | 78/88 (88.6) | 0.626 | 61/70 (87.1) | 68/74 (91.9) | 0.355 |  |
|  | | | | | | | | | | | |  |
| Any vaginal deliveries, n/N (%) |  | 141/160 (88.1) | 78/90 (86.7) | 61/68 (89.7) | 0.562 | 58/68 (85.3) | 80/89 (89.9) | 0.384 | 58/70 (82.9) | 68/75 (90.7) | 0.169* |  |
| Any cesarean deliveries, n/N (%) |  | 17/160 (10.6) | 9/90 (10.0) | 8/68 (11.8) | 0.723 | 9/68 (13.2) | 7/89 (7.9) | 0.275 | 7/70 (10.0) | 9/75 (12.0) | 0.701 |  |
|  | | | | | | | | | | | |  |
| Menopausal status, n/N (%) | Pre-menopausal | 14/160 (8.8) | 8/90 (8.9) | 6/68 (8.8) | 0.996 | 7/68 (10.3) | 7/89 (7.9) | 0.793 | 9/70 (12.9) | 5/75 (6.7) | 0.396 |  |
|  | Post-menopausal | 137/160 (85.6) | 77/90 (85.6) | 58/68 (85.3) |  | 57/68 (83.8) | 78/89 (87.6) |  | 56/70 (80.0) | 66/75 (88.0) |  |  |
|  | Not sure | 9/160 (5.6) | 5/90 (5.6) | 4/68 (5.9) |  | 4/68 (5.9) | 4/89 (4.5) |  | 5/70 (7.1) | 4/75 (5.3) |  |  |
|  | | | | | | | | | | | |  |
| Currently using estrogen, n/N (%) |  | 46/160 (28.8) | 25/90 (27.8) | 19/68 (27.9) | 0.982 | 13/68 (19.1) | 31/89 (34.8) | 0.032* | 14/70 (20.0) | 28/75 (37.3) | 0.023* |  |
|  | | | | | | | | | | | |  |
| Current smoker, n/N (%) |  | 13/160 (8.1) | 7/90 (7.8) | 6/68 (8.8) | 0.813 | 8/68 (11.8) | 5/89 (5.6) | 0.175* | 7/70 (10.0) | 4/75 (5.3) | 0.296 |  |
|  | | | | | | | | | | | |  |
| Urgency Urinary Incontinence, n/N (%) |  | 115/157 (73.2) | 65/90 (72.2) | 48/65 (73.8) | 0.823 | 50/68 (73.5) | 62/86 (72.1) | 0.843 | 49/70 (70.0) | 54/72 (75.0) | 0.506 |  |
|  | | | | | | | | | | | |  |
| Previous ABL surgery, n/N (%) |  | 8/160 (5.0) | 7/90 (7.8) | 1/68 (1.5) | 0.109* | 3/68 (4.4) | 5/89 (5.6) | 0.734 | 5/70 (7.1) | 3/75 (4.0) | 0.414 |  |
| Previous anal/rectal surgery, n/N (%) |  | 27/160 (16.9) | 17/90 (18.9) | 10/68 (14.7) | 0.490 | 13/68 (19.1) | 14/89 (15.7) | 0.578 | 12/70 (17.1) | 12/75 (16.0) | 0.853 |  |
| Previous UI surgery, n/N (%) |  | 40/160 (25.0) | 26/90 (28.9) | 13/68 (19.1) | 0.161* | 19/68 (27.9) | 20/89 (22.5) | 0.433 | 16/70 (22.9) | 21/75 (28.0) | 0.478 |  |
| Previous POP surgery, n/N (%) |  | 40/160 (25.0) | 25/90 (27.8) | 14/68 (20.6) | 0.301 | 15/68 (22.1) | 24/89 (27.0) | 0.481 | 15/70 (21.4) | 24/75 (32.0) | 0.154* |  |
| Hysterectomy, n/N (%) |  | 75/160 (46.9) | 42/90 (46.7) | 33/68 (48.5) | 0.816 | 33/68 (48.5) | 41/89 (46.1) | 0.759 | 26/70 (37.1) | 42/75 (56.0) | 0.024* |  |
|  | | | | | | | | | | | |  |
| Taking fiber supplements, n/N (%) |  | 65/155 (41.9) | 37/87 (42.5) | 28/66 (42.4) | 0.990 | 27/66 (40.9) | 37/86 (43.0) | 0.794 | 24/68 (35.3) | 37/72 (51.4) | 0.056* |  |
| Dietary fiber intake, mean (SD) [min, max], g |  | 13.9 (4.3) [4.9, 30.0] | 13.9 (4.3) [4.9, 30.0] | 13.8 (4.3) [5.6, 27.1] | 0.944 | 14.5 (4.4) [4.9, 30.0] | 13.4 (4.1) [5.6, 27.1] | 0.129* | 14.1 (4.3) [4.9, 30.0] | 14.0 (4.4) [5.6, 25.6] | 0.889 |  |
| Meat/Snack Screener Score, mean (SD) [min, max] |  | 18.3 (8.8) [0.0, 52.0] | 18.3 (9.3) [0.0, 52.0] | 18.4 (8.1) [2.0, 43.0] | 0.960 | 20.0 (10.2) [2.0, 52.0] | 17.2 (7.3) [0.0, 41.0] | 0.057* | 18.6 (9.5) [2.0, 52.0] | 17.7 (7.7) [0.0, 41.0] | 0.519 |  |
|  | | | | | | | | | | | |  |
| Bristol Stool Type, n/N (%) | Type 2 - Sausage-shaped but lumpy | 12/160 (7.5) | 5/90 (5.6) | 6/68 (8.8) | 0.506 | 6/68 (8.8) | 5/89 (5.6) | 0.656 | 5/70 (7.1) | 6/75 (8.0) | 0.282 |  |
|  | Type 3 - Like a sausage but with cracks on its surface | 22/160 (13.8) | 14/90 (15.6) | 7/68 (10.3) |  | 8/68 (11.8) | 13/89 (14.6) |  | 12/70 (17.1) | 10/75 (13.3) |  |  |
|  | Type 4 - Like a sausage or snake, smooth and soft | 42/160 (26.3) | 22/90 (24.4) | 20/68 (29.4) |  | 15/68 (22.1) | 26/89 (29.2) |  | 13/70 (18.6) | 26/75 (34.7) |  |  |
|  | Type 5 - Soft blobs with clear-cut edges | 33/160 (20.6) | 22/90 (24.4) | 11/68 (16.2) |  | 17/68 (25.0) | 16/89 (18.0) |  | 17/70 (24.3) | 13/75 (17.3) |  |  |
|  | Type 6 - Fluffy pieces with ragged edges, a mushy stool | 51/160 (31.9) | 27/90 (30.0) | 24/68 (35.3) |  | 22/68 (32.4) | 29/89 (32.6) |  | 23/70 (32.9) | 20/75 (26.7) |  |  |
|  | | | | | | | | | | | |  |
| Pain/discomfort in abdomen in last 3 months, n/N (%) | Less than once per week | 99/160 (61.9) | 55/90 (61.1) | 43/68 (63.2) | 0.786 | 42/68 (61.8) | 55/89 (61.8) | 0.997 | 39/70 (55.7) | 51/75 (68.0) | 0.129* |  |
|  | At least once per week | 61/160 (38.1) | 35/90 (38.9) | 25/68 (36.8) |  | 26/68 (38.2) | 34/89 (38.2) |  | 31/70 (44.3) | 24/75 (32.0) |  |  |
| Pain/discomfort 6 months or longer, n/N (%) |  | 79/160 (49.4) | 42/90 (46.7) | 36/68 (52.9) | 0.435 | 33/68 (48.5) | 45/89 (50.6) | 0.801 | 40/70 (57.1) | 30/75 (40.0) | 0.040* |  |
| Diagnosed with IBS, n/N (%) |  | 35/160 (21.9) | 16/90 (17.8) | 18/68 (26.5) | 0.279 | 13/68 (19.1) | 21/89 (23.6) | 0.793 | 15/70 (21.4) | 15/75 (20.0) | 0.943 |  |
| Frequency of loose/mushy/watery stools in last 3 months, n/N (%) | Never or rare | 55/160 (34.4) | 31/90 (34.4) | 23/68 (33.8) | 0.860 | 18/68 (26.5) | 35/89 (39.3) | 0.244 | 18/70 (25.7) | 33/75 (44.0) | 0.065* |  |
|  | Sometimes | 33/160 (20.6) | 20/90 (22.2) | 13/68 (19.1) |  | 16/68 (23.5) | 17/89 (19.1) |  | 16/70 (22.9) | 15/75 (20.0) |  |  |
|  | Often/most of the time/always | 72/160 (45.0) | 39/90 (43.3) | 32/68 (47.1) |  | 34/68 (50.0) | 37/89 (41.6) |  | 36/70 (51.4) | 27/75 (36.0) |  |  |
|  | | | | | | | | | | | |  |
| St. Mark's Score (start of run-in), mean (SD) [min, max] |  | 17.9 (2.6) [12.0, 24.0] | 18.2 (2.5) [12.0, 24.0] | 17.6 (2.7) [12.0, 23.0] | 0.146* | 18.5 (2.6) [12.0, 24.0] | 17.5 (2.4) [12.0, 22.0] | 0.019* | 18.2 (2.6) [12.0, 24.0] | 17.5 (2.5) [12.0, 23.0] | 0.118* |  |
|  | | | | | | | | | | | |  |
| Bowel movements per week (start of run-in), mean (SD) [min, max], No. |  | 12.7 (8.1) [0.0, 45.5] | 13.2 (8.7) [0.0, 45.5] | 12.0 (7.4) [1.0, 38.0] | 0.381 | 12.3 (7.5) [0.0, 43.8] | 13.1 (8.8) [0.0, 45.5] | 0.579 | 13.0 (9.2) [0.0, 45.5] | 12.5 (7.4) [0.0, 38.0] | 0.746 |  |
| Bowel movements with urgency per week (start of run-in), mean (SD) [min, max], No. |  | 6.0 (5.4) [0.0, 34.0] | 5.9 (5.2) [0.0, 26.5] | 6.0 (5.7) [0.0, 34.0] | 0.919 | 5.7 (4.5) [0.0, 21.9] | 6.2 (6.0) [0.0, 34.0] | 0.578 | 5.5 (4.8) [0.0, 21.9] | 6.3 (5.9) [0.0, 34.0] | 0.388 |  |
| Accident-free days per week (start of run-in), mean (SD) [min, max], No. |  | 3.0 (2.0) [0.0, 7.0] | 3.0 (2.1) [0.0, 7.0] | 3.1 (1.9) [0.0, 7.0] | 0.643 | 3.1 (2.1) [0.0, 7.0] | 2.9 (2.0) [0.0, 7.0] | 0.606 | 3.2 (2.1) [0.0, 7.0] | 3.0 (2.0) [0.0, 7.0] | 0.580 |  |
| Leaks per week (start of run-in), mean (SD) [min, max], No. |  | 8.1 (7.8) [0.0, 52.0] | 8.1 (7.2) [0.0, 36.5] | 8.0 (8.6) [0.0, 52.0] | 0.900 | 7.4 (6.0) [0.0, 26.0] | 8.7 (9.0) [0.0, 52.0] | 0.312 | 7.4 (7.6) [0.0, 39.0] | 7.9 (6.3) [0.0, 27.0] | 0.625 |  |
| Leaks with urgency per week (start of run-in), mean (SD) [min, max], No. |  | 3.4 (4.3) [0.0, 35.0] | 3.2 (3.9) [0.0, 25.0] | 3.5 (4.9) [0.0, 35.0] | 0.655 | 3.2 (3.0) [0.0, 14.0] | 3.5 (5.2) [0.0, 35.0] | 0.677 | 2.7 (2.5) [0.0, 10.0] | 3.3 (3.6) [0.0, 16.0] | 0.223 |  |
|  | | | | | | | | | | | |  |
| Patient Global Symptom Control, n/N (%) |  | 28/159 (17.6) | 16/89 (18.0) | 11/68 (16.2) | 0.767 | 12/68 (17.6) | 14/88 (15.9) | 0.773 | 13/70 (18.6) | 11/74 (14.9) | 0.552 |  |
|  | | | | | | | | | | | |  |

FIE = fecal incontinence episode; ABL = accidental bowel leakage; IBS = irritable bowel syndrome; UI = urinary incontinence; POP = pelvic organ prolapse; SD = standard deviation;

*indicates factor included in multivariable model prior to backward selection given p<0.2

^a^ P-values from bivariate tests come from chi-square tests, Student’s t-tests, and Wilcoxon rank sum tests, as appropriate.

^b^ Participants were able to select American Indian/Alaska Native, Asian, or more than one race as well as an “Other” race category, which was accompanied by a free response field. Due to the small numbers of participants, these categories were combined for the purposes of this analysis.
