# Supplementary material for: Characteristics associated with subjective and objective measures of treatment success in women undergoing percutaneous tibial nerve stimulation vs sham for accidental bowel leakage
Source: Int Urogynecol J. 2023 Jan 27;34(8):1715–23. doi: 10.1007/s00192-022-05431-y (PMC10372194; doi:10.1007/s00192-022-05431-y)
Supplement: Supplementary file 2 — (DOCX 54 kb) [file 192_2022_5431_MOESM2_ESM.docx]

**Supplemental Table 2. Characteristics of Women with Success vs Failure with varying change from baseline St Mark’s Score**

|  | | | ***Responder Status (>= 3-point decrease)*** | | | ***Responder Status (>= 5-point decrease)*** | | |
| --- | --- | --- | --- | --- | --- | --- | --- | --- |
| ***Characteristic*** | ***Category*** | ***Total (N=158)*** | ***Responder (N=102)*** | ***Non-responder (N=56)*** | ***P-value*** | ***Responder (N=73)*** | ***Non-responder (N=85)*** | ***P-value*** |
| Randomized Treatment, n/N (%) | PTNS | 104/158 (65.8) | 68/102 (66.7) | 36/56 (64.3) | 0.7628 | 52/73 (71.2) | 52/85 (61.2) | 0.1852* |
|  | Sham | 54/158 (34.2) | 34/102 (33.3) | 20/56 (35.7) |  | 21/73 (28.8) | 33/85 (38.8) |  |
|  | | | | | |  |  |  |
| Age, mean (SD) [min, max], y |  | 63.7 (11.5) [29.0, 90.0] | 64.3 (11.2) [29.0, 85.0] | 62.7 (12.1) [34.0, 90.0] | 0.4107 | 63.7 (12.2) [29.0, 85.0] | 63.8 (10.9) [34.0, 90.0] | 0.9387 |
|  | | | | | |  |  |  |
| Race, n/N (%) | American Indian or Alaska Native | 2/158 (1.3) | 2/102 (2.0) | 0/56 (0.0) | 0.9988 | 0/73 (0.0) | 2/85 (2.4) | 0.9828 |
|  | Asian | 2/158 (1.3) | 2/102 (2.0) | 0/56 (0.0) |  | 1/73 (1.4) | 1/85 (1.2) |  |
|  | Black or African American | 18/158 (11.4) | 12/102 (11.8) | 6/56 (10.7) |  | 10/73 (13.7) | 8/85 (9.4) |  |
|  | Native Hawaiian or Other Pacific Islander | 2/158 (1.3) | 0/102 (0.0) | 2/56 (3.6) |  | 0/73 (0.0) | 2/85 (2.4) |  |
|  | White | 127/158 (80.4) | 82/102 (80.4) | 45/56 (80.4) |  | 58/73 (79.5) | 69/85 (81.2) |  |
|  | More than one race | 3/158 (1.9) | 2/102 (2.0) | 1/56 (1.8) |  | 2/73 (2.7) | 1/85 (1.2) |  |
|  | Unknown/Not Reported | 4/158 (2.5) | 2/102 (2.0) | 2/56 (3.6) |  | 2/73 (2.7) | 2/85 (2.4) |  |
|  | | | | | |  |  |  |
| Ethnicity, n/N (%) | Hispanic/Latina | 14/158 (8.9) | 10/102 (9.8) | 4/56 (7.1) | 0.8458 | 8/73 (11.0) | 6/85 (7.1) | 0.6783 |
|  | Not Hispanic/Latina | 143/158 (90.5) | 91/102 (89.2) | 52/56 (92.9) |  | 64/73 (87.7) | 79/85 (92.9) |  |
|  | Unknown/Not Reported | 1/158 (0.6) | 1/102 (1.0) | 0/56 (0.0) |  | 1/73 (1.4) | 0/85 (0.0) |  |
|  | | | | | |  |  |  |
| Primary language, n/N (%) | English | 153/158 (96.8) | 99/102 (97.1) | 54/56 (96.4) | 0.8289 | 70/73 (95.9) | 83/85 (97.6) | 0.5346 |
|  | Spanish | 5/158 (3.2) | 3/102 (2.9) | 2/56 (3.6) |  | 3/73 (4.1) | 2/85 (2.4) |  |
|  | | | | | |  |  |  |
| Education, n/N (%) | Some college or greater | 109/158 (69.0) | 76/102 (74.5) | 33/56 (58.9) | 0.0445* | 54/73 (74.0) | 55/85 (64.7) | 0.2107 |
|  | No college education | 49/158 (31.0) | 26/102 (25.5) | 23/56 (41.1) |  | 19/73 (26.0) | 30/85 (35.3) |  |
|  | | | | | |  |  |  |
| Insurance status, n/N (%) | Private/HMO | 57/158 (36.1) | 37/102 (36.3) | 20/56 (35.7) | 0.9064 | 26/73 (35.6) | 31/85 (36.5) | 0.2990 |
|  | Medicare/Medicaid | 43/158 (27.2) | 29/102 (28.4) | 14/56 (25.0) |  | 24/73 (32.9) | 19/85 (22.4) |  |
|  | Both Private and Medicare/Medicaid | 40/158 (25.3) | 24/102 (23.5) | 16/56 (28.6) |  | 14/73 (19.2) | 26/85 (30.6) |  |
|  | Other/None | 18/158 (11.4) | 12/102 (11.8) | 6/56 (10.7) |  | 9/73 (12.3) | 9/85 (10.6) |  |
|  | | | | | |  |  |  |
| Body Mass Index, mean (SD) [min, max] |  | 29.4 (6.6) [18.0, 55.0] | 28.3 (5.7) [18.0, 44.0] | 31.5 (7.6) [21.0, 55.0] | 0.0051* | 28.5 (5.8) [19.0, 44.0] | 30.2 (7.2) [18.0, 55.0] | 0.1277* |
| Body Mass Index, n/N (%) | <25 kg/m2 | 38/156 (24.4) | 30/100 (30.0) | 8/56 (14.3) | 0.0193* | 20/73 (27.4) | 18/83 (21.7) | 0.0234* |
|  | 25 - 29.9 kg/m2 | 51/156 (32.7) | 35/100 (35.0) | 16/56 (28.6) |  | 30/73 (41.1) | 21/83 (25.3) |  |
|  | >= 30 kg/m2 | 67/156 (42.9) | 35/100 (35.0) | 32/56 (57.1) |  | 23/73 (31.5) | 44/83 (53.0) |  |
|  | | | | | |  |  |  |
| Anal sphincter squeeze, n/N (%) |  | 139/156 (89.1) | 90/100 (90.0) | 49/56 (87.5) | 0.6314 | 66/73 (90.4) | 73/83 (88.0) | 0.6235 |
|  | | | | | |  |  |  |
| Any vaginal deliveries, n/N (%) |  | 139/158 (88.0) | 89/102 (87.3) | 50/56 (89.3) | 0.7077 | 63/73 (86.3) | 76/85 (89.4) | 0.5500 |
| Any cesarean deliveries, n/N (%) |  | 17/158 (10.8) | 10/102 (9.8) | 7/56 (12.5) | 0.6017 | 9/73 (12.3) | 8/85 (9.4) | 0.5563 |
|  | | | | | |  |  |  |
| Menopausal status, n/N (%) | Pre-menopausal | 14/158 (8.9) | 8/102 (7.8) | 6/56 (10.7) | 0.8293 | 8/73 (11.0) | 6/85 (7.1) | 0.6937 |
|  | Post-menopausal | 135/158 (85.4) | 88/102 (86.3) | 47/56 (83.9) |  | 61/73 (83.6) | 74/85 (87.1) |  |
|  | Not sure | 9/158 (5.7) | 6/102 (5.9) | 3/56 (5.4) |  | 4/73 (5.5) | 5/85 (5.9) |  |
|  | | | | | |  |  |  |
| Currently using estrogen, n/N (%) |  | 44/158 (27.8) | 27/102 (26.5) | 17/56 (30.4) | 0.6024 | 21/73 (28.8) | 23/85 (27.1) | 0.8110 |
|  | | | | | |  |  |  |
| Current smoker, n/N (%) |  | 13/158 (8.2) | 7/102 (6.9) | 6/56 (10.7) | 0.4029 | 7/73 (9.6) | 6/85 (7.1) | 0.5653 |
|  | | | | | |  |  |  |
| Urgency Urinary Incontinence, n/N (%) |  | 113/155 (72.9) | 72/102 (70.6) | 41/53 (77.4) | 0.3696 | 54/73 (74.0) | 59/82 (72.0) | 0.7779 |
|  | | | | | |  |  |  |
| Previous ABL surgery, n/N (%) |  | 8/158 (5.1) | 7/102 (6.9) | 1/56 (1.8) | 0.1961* | 6/73 (8.2) | 2/85 (2.4) | 0.1150* |
| Previous anal/rectal surgery, n/N (%) |  | 27/158 (17.1) | 17/102 (16.7) | 10/56 (17.9) | 0.8492 | 27/158 (17.1) | 15/73 (20.5) | 12/85 (14.1) |
| Previous UI surgery, n/N (%) |  | 39/158 (24.7) | 28/102 (27.5) | 11/56 (19.6) | 0.2783 | 39/158 (24.7) | 24/73 (32.9) | 15/85 (17.6) |
| Previous POP surgery, n/N (%) |  | 39/158 (24.7) | 28/102 (27.5) | 11/56 (19.6) | 0.2783 | 39/158 (24.7) | 21/73 (28.8) | 18/85 (21.2) |
| Hysterectomy, n/N (%) |  | 75/158 (47.5) | 47/102 (46.1) | 28/56 (50.0) | 0.6369 | 75/158 (47.5) | 34/73 (46.6) | 41/85 (48.2) |
|  | | | | | |  |  |  |
| Taking fiber supplements, n/N (%) |  | 65/153 (42.5) | 42/99 (42.4) | 23/54 (42.6) | 0.9839 | 30/71 (42.3) | 35/82 (42.7) | 0.9573 |
| Dietary fiber intake, mean (SD) [min, max], g |  | 13.9 (4.3) [4.9, 30.0] | 13.9 (4.1) [4.9, 30.0] | 13.8 (4.6) [5.6, 27.1] | 0.9769 | 13.9 (4.1) [4.9, 25.6] | 13.8 (4.5) [5.6, 30.0] | 0.9917 |
| Meat/Snack Screener Score, mean (SD) [min, max] |  | 18.4 (8.8) [0.0, 52.0] | 17.9 (9.2) [0.0, 52.0] | 19.2 (7.9) [4.0, 43.0] | 0.3896 | 17.7 (9.5) [0.0, 52.0] | 18.9 (8.1) [2.0, 43.0] | 0.4195 |
|  | | | | | |  |  |  |
| Bristol Stool Type, n/N (%) | Type 2 - Sausage-shaped but lumpy | 11/158 (7.0) | 6/102 (5.9) | 5/56 (8.9) | 0.9651 | 5/73 (6.8) | 6/85 (7.1) | 0.4507 |
|  | Type 3 - Like a sausage but with cracks on its surface | 21/158 (13.3) | 14/102 (13.7) | 7/56 (12.5) |  | 13/73 (17.8) | 8/85 (9.4) |  |
|  | Type 4 - Like a sausage or snake, smooth and soft | 42/158 (26.6) | 27/102 (26.5) | 15/56 (26.8) |  | 16/73 (21.9) | 26/85 (30.6) |  |
|  | Type 5 - Soft blobs with clear-cut edges | 33/158 (20.9) | 22/102 (21.6) | 11/56 (19.6) |  | 17/73 (23.3) | 16/85 (18.8) |  |
|  | Type 6 - Fluffy pieces with ragged edges, a mushy stool | 51/158 (32.3) | 33/102 (32.4) | 18/56 (32.1) |  | 22/73 (30.1) | 29/85 (34.1) |  |
|  | | | | | |  |  |  |
| Pain/discomfort in abdomen in last 3 months, n/N (%) | Less than once per week | 98/158 (62.0) | 65/102 (63.7) | 33/56 (58.9) | 0.5526 | 43/73 (58.9) | 55/85 (64.7) | 0.4541 |
|  | At least once per week | 60/158 (38.0) | 37/102 (36.3) | 23/56 (41.1) |  | 30/73 (41.1) | 30/85 (35.3) |  |
| Pain/discomfort 6 months or longer, n/N (%) |  | 78/158 (49.4) | 48/102 (47.1) | 30/56 (53.6) | 0.4339 | 34/73 (46.6) | 44/85 (51.8) | 0.5156 |
| Diagnosed with IBS, n/N (%) |  | 34/158 (21.5) | 17/102 (16.7) | 17/56 (30.4) | 0.1394* | 11/73 (15.1) | 23/85 (27.1) | 0.1010* |
| Frequency of loose/mushy/watery stools in last 3 months, n/N (%) | Never or rare | 54/158 (34.2) | 38/102 (37.3) | 16/56 (28.6) | 0.5424 | 25/73 (34.2) | 29/85 (34.1) | 0.5066 |
|  | Sometimes | 33/158 (20.9) | 20/102 (19.6) | 13/56 (23.2) |  | 18/73 (24.7) | 15/85 (17.6) |  |
|  | Often/most of the time/always | 71/158 (44.9) | 44/102 (43.1) | 27/56 (48.2) |  | 30/73 (41.1) | 41/85 (48.2) |  |
|  | | | | | |  |  |  |
| St. Mark's Score (start of run-in), mean (SD) [min, max] |  | 17.9 (2.6) [12.0, 24.0] | 18.2 (2.4) [12.0, 24.0] | 17.4 (2.7) [12.0, 23.0] | 0.0526* | 18.3 (2.4) [12.0, 24.0] | 17.6 (2.6) [12.0, 23.0] | 0.0629* |
|  | | | | | |  |  |  |
| Bowel movements per week (start of run-in), mean (SD) [min, max], No. |  | 12.7 (8.2) [0.0, 45.5] | 13.2 (8.4) [0.0, 45.5] | 11.7 (7.8) [1.0, 38.0] | 0.2762 | 13.3 (8.2) [0.0, 43.8] | 12.3 (8.2) [0.0, 45.5] | 0.4537 |
| Bowel movements with urgency per week (start of run-in), mean (SD) [min, max], No. |  | 6.0 (5.4) [0.0, 34.0] | 6.1 (5.1) [0.0, 26.5] | 5.6 (5.9) [0.0, 34.0] | 0.5676 | 6.2 (5.5) [0.0, 26.5] | 5.8 (5.3) [0.0, 34.0] | 0.6397 |
| Accident-free days per week (start of run-in), mean (SD) [min, max], No. |  | 3.0 (2.0) [0.0, 7.0] | 3.1 (2.1) [0.0, 7.0] | 2.9 (1.9) [0.0, 7.0] | 0.5681 | 3.1 (2.0) [0.0, 7.0] | 2.9 (2.0) [0.0, 7.0] | 0.5582 |
| Leaks per week (start of run-in), mean (SD) [min, max], No. |  | 8.0 (7.8) [0.0, 52.0] | 7.8 (7.0) [0.0, 36.5] | 8.5 (9.3) [0.0, 52.0] | 0.5723 | 7.0 (5.4) [0.0, 22.0] | 9.0 (9.4) [0.0, 52.0] | 0.1320* |
| Leaks with urgency per week (start of run-in), mean (SD) [min, max], No. |  | 3.4 (4.4) [0.0, 35.0] | 3.1 (3.7) [0.0, 25.0] | 3.8 (5.4) [0.0, 35.0] | 0.3335 | 3.0 (3.2) [0.0, 14.0] | 3.6 (5.2) [0.0, 35.0] | 0.4065 |
|  | | | | | |  |  |  |
| Patient Global Symptom Control, n/N (%) |  | 27/157 (17.2) | 18/101 (17.8) | 9/56 (16.1) | 0.7808 | 14/73 (19.2) | 13/84 (15.5) | 0.5405 |
|  | | | | | |  |  |  |

FIE = fecal incontinence episode; ABL = accidental bowel leakage; IBS = irritable bowel syndrome; UI = urinary incontinence; POP = pelvic organ prolapse; SD = standard deviation;

*indicates factor included in multivariable model prior to backward selection given p<0.2

^a^ P-values from bivariate tests come from chi-square tests, Student’s t-tests, and Wilcoxon rank sum tests, as appropriate.

^b^ Participants were able to select American Indian/Alaska Native, Asian, or more than one race as well as an “Other” race category, which was accompanied by a free response field. Due to the small numbers of participants, these categories were combined for the purposes of this analysis.
