# Supplementary material for: Characteristics associated with subjective and objective measures of treatment success in women undergoing percutaneous tibial nerve stimulation vs sham for accidental bowel leakage
Source: Int Urogynecol J. 2023 Jan 27;34(8):1715–23. doi: 10.1007/s00192-022-05431-y (PMC10372194; doi:10.1007/s00192-022-05431-y)
Supplement: Supplementary file 3 — (DOCX 42 kb) [file 192_2022_5431_MOESM3_ESM.docx]

**Supplemental Table 3. Multivariable Logistic Regression Model Predicting Treatment Success with varying change from baseline St Mark’s Score**

|  | | | ***Odds Ratio (95% Confidence Interval) ^b^*** | | | | ***Observed Proportion  for each comparison n/N (%) ^a^*** | |
| --- | --- | --- | --- | --- | --- | --- | --- | --- |
| ***Model Effect*** | ***Model p-value*** | ***Comparison (1 vs 2)*** | ***Estimate*** | ***Lower Bound*** | ***Upper Bound*** | ***Comparison p-value*** | ***1*** | ***2*** |
| **3-point reduction from Baseline St. Mark’s Score** | | | | | | | | |
| Site | 0.6232 |  | . | . | . |  |  |  |
| St. Mark's Score (start of run-in) | 0.0574 |  | 1.155 | 0.995 | 1.340 | 0.0574 |  |  |
| Body Mass Index (categorical) | 0.0402 | <25 kg/m2 vs 25 - 29.9 kg/m2 | 2.079 | 0.700 | 6.172 | 0.1875 | 30/38 (79) | 35/51 (69) |
|  |  | <25 kg/m2 vs >= 30 kg/m2 | 3.723 | 1.318 | 10.522 | 0.0131* | 30/38 (79) | 35/67 (52) |
|  |  | 25 - 29.9 kg/m2 vs >= 30 kg/m2 | 1.791 | 0.770 | 4.167 | 0.1762 | 35/51 (69) | 35/67 (52) |
| **5-point reduction from Baseline St. Mark’s Score** | | | | | | | | |
| Site | 0.0776 |  | . | . | . |  |  |  |
| St. Mark's Score (start of run-in) | 0.0120 |  | 1.221 | 1.045 | 1.428 | 0.0120* |  |  |
| Body Mass Index (categorical) | 0.0779 | <25 kg/m2 vs 25 - 29.9 kg/m2 | 0.891 | 0.317 | 2.500 | 0.8260 | 20/38 (53) | 30/51 (59) |
|  |  | <25 kg/m2 vs >= 30 kg/m2 | 2.337 | 0.840 | 6.501 | 0.1040 | 20/38 (53) | 23/67 (34) |
|  |  | 25 - 29.9 kg/m2 vs >= 30 kg/m2 | 2.624 | 1.072 | 6.419 | 0.0346* | 30/51 (59) | 23/67 (34) |
| Previous UI surgery | 0.0199 | Yes vs No | 3.086 | 1.196 | 7.968 | 0.0199* | 24/39 (62) | 49/119 (41) |
| Leaks per week (start of run-in) | 0.0081 |  | 0.919 | 0.863 | 0.978 | 0.0081* |  |  |

FIE = fecal incontinence episode; ABL = accidental bowel leakage; IBS = irritable bowel syndrome; UI = urinary incontinence; POP = pelvic organ prolapse; SD = standard deviation;

* Indicates statistically significant effect in the model.

^a^ Observed proportions of success [n/N (%)] are presented for categorical variables interactions.

^b^ Odds ratios calculated from backward-selected model and are adjusted for the selected risk factors. Odds ratios for risk factors included in interactions were calculated within each level of the other interaction variable.
